# Safety Assessment of One *Lactiplantibacillus plantarum* Isolated from the Traditional Chinese Fermented Vegetables—Jiangshui

**DOI:** 10.3390/foods11152177

**Published:** 2022-07-22

**Authors:** Dexin Ou, Na Ling, Xihao Wang, Yanyan Zou, Jingjing Dong, Danfeng Zhang, Yizhong Shen, Yingwang Ye

**Affiliations:** School of Food and Biological Engineering, Hefei University of Technology, Hefei 230009, China; onlyodx@sina.com (D.O.); lingna02@163.com (N.L.); wang1140305@gmail.com (X.W.); zouyanyan7844@163.com (Y.Z.); dongjingjing0520@163.com (J.D.); zhangdanfeng@hfut.edu.cn (D.Z.); yzshen@hfut.edu.cn (Y.S.)

**Keywords:** *Lactiplantibacillus plantarum*, probiotic, safety assessment, toxicity test

## Abstract

*Lactiplantibacillus plantarum* is a kind of extensively utilized probiotic species, which plays a critical role in the prevention of pathogenic bacteria and development of functional probiotics. Our group previously isolated one *Lactiplantibacillus* from Jiang Shui, a traditional Chinese fermented vegetable, which remarkably inhibited the growth of *Aspergillus flavus*. Herein, the safety of this isolate was assessed to ensure its application feasibility in food industry. Firstly, the phenotypic analyses including tolerance to low pH and bile salt, aggregation ability, and hemolytic activity detection, indicated the isolate could survive and colonize in the gastrointestinal tract, without hemolysin activity. The susceptibilities of the isolate to eight antibiotics and the absence of most resistance genes were demonstrated by agar disk diffusion and PCR, respectively. Furthermore, no mortality or toxicity was observed in mice by in vivo tests using gross autopsy, hematology, serum biochemistry, and HE-staining. Taken together, this study demonstrated the safety of *Lactiplantibacillus plantarum* WYH as a probiotic strain in terms of phenotypic analyses, absence of antimicrobial resistance and toxin-related genes, as well as mice toxicity test, while supported the prospect of applying isolate in suppression of fungal growth and mycotoxin biosynthesis.

## 1. Introduction

The term “Probiotics” was first proposed by Park in 1974 as “microorganisms or substances that improve the intestinal balance of host animals” [1]. In 2002, Food and Agriculture Organization (FAO) and World Health Organization (WHO) introduced the final universal definition of probiotics as living microorganisms that, when provided adequately, promote the host’s health [2]. The human gastrointestinal system is believed to contain 10–100 trillion microorganisms, some of which are probiotics, and they perform health-promoting biological actions by stabilizing the intestinal microbial environment and enhancing the intestinal permeability barrier [3]. Simultaneously, substantial research on probiotics has revealed that they have beneficial effects on illness prevention, intestinal management, immune function, and disease therapy [4]. *Lactiplantibacillus plantarum* (*L. plantarum*), a gram-like facultative anaerobe, is a kind of Generally Recognized as Safe (GRAS) [2]. It was commonly found in handmade fermented foods for a long history of human consumption and was always applicated in food preservation and probiotic usage [5]. As one of the important probiotics of human gut microbiota, *L. plantarum*, like others with various physiological functions, also exhibits superior efficacy in antibacterial and binding of various toxins [6,7].

However, recent findings have identified potential safety risks with part of GRAS strains. European Food Safety Authority (EFSA) has registered sepsis cases caused by *Lactiplantibacillus rhamnosus* during probiotic therapy, and endocarditis caused by *Lactiplantibacillus* can also lead to high mortality, thus we should emphasize the evaluation of the safety of edible microorganisms [8,9]. Our previous studies had found that the novel isolate had a marked inhibitory effect on the growth of *Aspergillus flavus*. Aflatoxins are the best-known secondary metabolites that have a potent carcinogenic produced by the *Aspergillus* species. The contamination of it in crops was prevalent, threatening human health and causing serious economic losses [10]. The availability of a generally safe and effective non-aflatoxigenic strain is vital for an economical and environmentally friendly contamination control method.

The safety of starter cultures, such as *L. plantarum*, is usually assumed based on the history of consuming large quantities of fermented foods, without experimental tests [9]. To exploit its potential biocontrol capability in the food industry, a series of tests were conducted to verify its safety. Firstly, in vitro tests were carried out aiming to analyze its viability in the gastrointestinal tract, hemolytic activities, and aggregation properties. Then, agar disk diffusion and PCR were performed to determine the antibiotic resistance and the existence of resistance genes of this strain. Finally, 14-day acute toxicity testing with single doses and 28-day repeated experiments with different dosages were conducted to analyze toxicity in vivo.

## 2. Materials and Methods

### 2.1. Experimental Animals

Male and female Kunming mice (18–22 g clean level) were purchased from Anhui Medical University (Hefei, China). After seven days of acclimatized feeding in a controlled environment and humidity (25 ± 2 °C, 60 ± 10%), with a constant light/dark cycle of 12 h and enough water and feed. After proper labeling, the mice were randomly assigned different marked cages. All the animal experiments were conducted with the approval of the institutional animal care and use committee of the Hefei University of Technology (Hefei, China), and the project identification code is HFUT20210224001 (24 February 2021).

### 2.2. Preparation of L. Plantarum WYH

*L. plantarum* WYH strain was isolated from the traditional Chinese fermented vegetables, Jiangshui, and stored in our laboratory. The isolate was identified by 16S rRNA gene sequence analysis and the raw data were deposited in the NCBI database (serial number: SUB8727504). The isolates were cultured separately in an MRS broth medium (Huankai, Guangzhou, China) for 16 h at 37 °C and then centrifuged at 5000× *g* for 10 min. After discarding the supernatants and washing three times with normal saline, the cell pellets were placed in the sterile 0.9% sodium chloride solution and freshly prepared daily before feeding the animals.

### 2.3. Antibiotic Susceptibility Test

Overnight strains were inoculated into fresh MRS and cultured to an optical density at 600 nm (OD_600_) reached 0.8. Then, cells were diluted to 10^5^–10^6^ CFU/mL in sterile 0.9% NaCl and homogeneously layered on the surface of the MRS agar with 100 μL. Sterile disks (5.0 mm diameter) were affixed to the plate in triplicate and incubated at 37 °C for 16–18 h. Sensitivity to antibiotics was per CLSI guidelines determined by the appearance and the diameters of the transparent circles on the media and was shown as the mean from three duplicates [11].

### 2.4. Detecting Antibiotic Resistance Genes

Total genomic DNA was extracted using a DNA extraction Kit (Sangon, Shanghai, China) following the instructions. Primers to be used in the detection of the isolate were selected from all the classes of antibiotics from published studies (Table 1). All amplified PCR products were analyzed on 1% agarose gels and stained with ethidium bromide [12].

### 2.5. Study on Acid Resistance and Bile Salt Tolerance

Different concentrations of acid (pH 4.5, 3.5, 2.5) or bile salt (0.2, 0.4, 0.8%) solution and 50 µL of the culture (10^8^ CFU/mL) were added to test tubes containing 4.95 mL of MRS media. A total of 200 µL of each mixture was inoculated into 96-wells in triplicate and grown at 37 °C microaerophilic conditions. The OD_600_ was measured after 16 h. To determine bile salt tolerance of the isolate after pre-exposure to low pH, the overnight strain was harvested by centrifugation (5000× *g*, 10 min) and resuspended into MRS broth adjusted to pH 3.5/2.5. After incubation for 3 h at 37 °C, cells were harvested and inoculated into MRS broth with different concentrations of bile salts (0, 0.1, 0.2, 0.3, 0.4%) at 37 °C for 16 h. Then, measured the absorbance at 600 nm. The results shown were pooled from three biological replicates.

### 2.6. Auto-Aggregation and Co-Aggregation Ability

Overnight LAB cells were harvested and resuspended in PBS solution after washing three times. The ability of aggregation was evaluated according to Dlamini and Escamilla-Montes with some modifications [13,14]. In this section, *Escherichia coli* ATCC25922 (gram-negative, *E. coli*) and *Staphylococcus aureus* ATCC25923 (gram-positive, *S. aureus*) were used as control cultures and the same sample pretreatment method as previously. The OD_600_ was measured separately at 0, 1, 2, and 3 h while the three strains were alone and at a 1:1 mixture. Agg% = [1 − (OD_600_ of upper suspension at time t/OD_600_ of total bacterial suspension at time 0)] × 100 in different time periods.

### 2.7. Hemolytic Assay

To explore the hemolysis, the isolated strain and the quality control strain *S. aureus* ATCC25923 were inoculated on a Columbia blood AGAR plate using a sterilized inoculation ring. The hemolytic zones were observed in 48 h of cultivation at 37 °C. Independent experiments were performed in triplicate.

### 2.8. In Vivo Toxicity Experiments (Acute Oral Toxicity and Repeated Dose 28-Day Oral Toxicity Study)

In vivo experiments were under the guidance of OECD and National Standards of the People’s Republic of China [15,16,17,18]. All the animal experiments were performed following the GB15193.3–2014 GB15193.22–2014. For acute oral toxicity, fresh *L. plantarum* was diluted in sterile 0.9% NaCl to 10^11^ CFU/mL every day. A total of 20 mice (half male and female) were randomly divided into two groups and treated with saline or *L. plantarum* 0.2 mL orally every day at the same time of day after fasting for 4 h [19]. One hour after administration, the animals were provided with food and water, then were observed for signs of clinical symptoms and mortality. Every three days, record body weight (BW) and food consumption (FC). After 14 days of treatment, animals fasted for at least 16 h before they were euthanized with pentobarbital sodium, and blood samples were collected. For the 28-day oral toxicity test, the basic treatment was the same as the acute oral toxicity test, the difference was the duration of the gavage and the concentration of the bacterial cultures. There will be four groups, a normal control group (NC), a low-dose group (LDG), a medium-dose group (MDG), and a high-dose group (HDG), in this procedure and each group contains 20 mice (equally male and female). The NC was fed with the saline and LDG, MDG, and HDG were fed with the saline with 10^8^, 10^9^, and 10^10^ CFU/mL of isolate, respectively. In addition to a hematological examination, serological analysis was performed. Mice were executed by the spinal dislocation method and the spleen, heart, liver, and kidney were weighted and calculated as the relative weight. The blood samples were collected for hematological parameters. For histological examination, the liver, kidney, and intestinal tract tissues were sectioned and embedded, and then stained with hematoxylin and eosin (HE). A complete blood count was evaluated by a Mindray BC-5000 Vet Auto Haematology Analyser (Shenzhen, China) based on hematology parameters. The serum biochemical parameters have been used in the kits and tested by an automatic biochemical analyzer (Rayto, Shenzhen, China).

### 2.9. Statistical Analysis

Statistical analysis was performed with GraphPad 8.0 software (San Diego, CA, USA). The data were performed with unpaired Student’s t-test or a one-way analysis of variance (ANOVA) with Dunnett’s post-test when appropriate. Mean values reported with standard error mean (mean ± SEM) were used to express all values unless otherwise indicated as appropriate. The *p* < 0.05 values denote significant differences among groups.

## 3. Results and Discussion

### 3.1. Tolerance to Acid and Bile Salts

**Figure 1:**
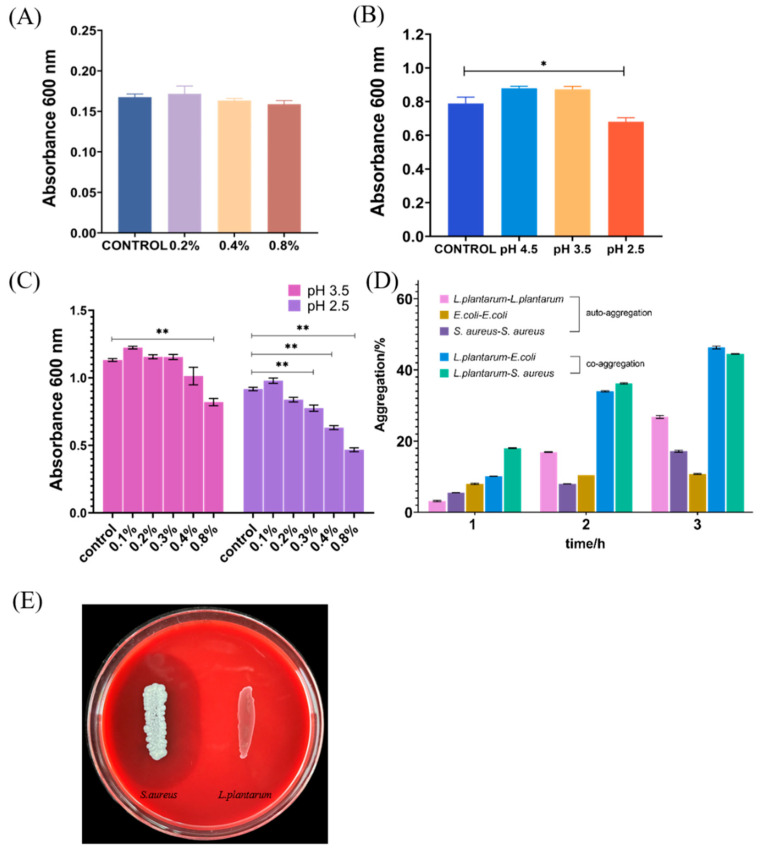
Absorbance of *L. plantarum* after incubation for 16 h in MRS broth at 0.2%, 0.4%, 0.8% bile salts (**A**) and pH 2.5, 3.5, 4.5 (**B**). (**C**) The absorbance of *L. plantarum* after incubation in different bile salts concentrations of the isolate after pre-exposure to low pH. (**D**) Auto-aggregation and co-aggregation with *S. aureus* and *E. coli* as determined after various times of incubation durations at 37 °C. (**E**) Hemolytic activity. Each experiment was independently repeated three times. * *p* < 0.05, ** *p* < 0.01.

Bile salt and acid tolerance are essential properties of *Lactiplantibacillus* strains, as bacteria cannot maintain integrated membrane structure at a high concentration of bile salts and require this ability to survive, proliferate, and sustain energy during gastrointestinal transit [20,21]. In the study shown in Figure 1A, the isolate exhibited a good growth ability in different concentrations of bile salts and showed no difference in all groups, even the highest concentration group (0.8%) exhibited the same trend in growth, indicating the strain had superiority to bile salt tolerance and could maintain viability in the presence of high concentration bile salts. Furthermore, significant differences were observed in growth between the control and pH 2.5 groups, but the growing ratio was still kept at 86.2% (Figure 1B), suggesting that the isolate can maintain good vitality in gastric acid. In this case, the bile salts tolerance of the isolate after pre-exposure to low pH was explored, which was simulated digestive processes. The results showed that the growth ability in 0.8% bile salt after pre-exposure to pH 2.5/3.5 was remarkably reduced, but the ratio of all 0.8% bile salt groups with control (without bile salts) maintain greater than half (Figure 1C). Except for this, in group pH 3.5, the growth ability was not remarkedly affected by various bile salts concentrations, offering a promising potential for screening of probiotics. Considering the mean bile concentration of the human gastrointestinal tract can be assessed as 0.03–0.3% *w/v* [22], the growth percentage in group pH 3.5 still exceeded 50% compared with the control (Figure 1C), implying the novel isolate can be considered to show good tolerance in the intestinal tract. A similar feature also existed in the strain isolated from yaks and fermented olives, which can mostly maintain a higher vitality when exposed to pH 2.5–4.0 and 0.3–0.5% bile salt [22].

### 3.2. Auto-Aggregation and Co-Aggregation

Auto-aggregation and co-aggregation are some of the abilities of probiotics to persist in the intestinal tract and adhere to mucus and epithelial cells in the host, which are utilized for screening the potential probiotics [23,24]. As depicted in Figure 1D, the novel isolates exhibited a superior auto-aggregation at 2 h and 3 h compared to the *E. coli* and *S. aureus*, and the rate of it was nearly half of the others at 2 h, which provided the prerequisite ability for the isolate to intestinal epithelial cells. The observed results showed the ability of co-aggregation of the *L. plantarum* isolates to *E. coli* or *S. aureus* has dramatically increased after 1 h. In pathogenic bacteria, aggregation was usually associated with pathogenesis mechanisms, as the formation of microcolonies may lead to an effective increase in the concentration of effectors secreted in or near the host cell, thereby regulating toxicity [23]. Conversely, the aggregation ability of probiotics, which is needed for probiotics to survive in the host intestines, is also an effective protective mechanism, reducing greatly the colonization of pathogenic bacteria after its aggregation with pathogenic bacteria on the surface [25]. Figure 1D also showed the 3 h auto-aggregation rate was nearly 30%, and the co-aggregation efficiency was nearly 50%, implying it can adhere to the intestinal epithelial cells, which facilitated the probiotics to better perform their probiotic effects. Among the three strains of *Lactiplantibacillus* isolated from *pericarpium citri reticulatae* by He, the self-aggregation rate at 4 h was 15–45%, while the co-aggregation rate for pathogenic bacteria such as *S. aureus* and *E. coli* was probably between 45–60% and these *Lactiplantibacillus* were also confirmed to obtain cholesterol-lowering potential and carbohydrate utilization capability in subsequent experiments [26].

### 3.3. Hemolysis Activity

Red blood cells and hemolysin (an immunogenic novel toxin with hemolytic), when activated by complement, can dissolve the antibody and then damage the red blood cells, resulting in a cascade of events such as anemia [27,28]. To determine the type of hemolysis, blood agar plates were used. The clear zones around colonies mean α-hemolysis, the green-hued zones around colonies mean β-hemolysis, and no zones around colonies mean γ-hemolysis. The isolate exhibited no visible hemolysis on the blood agar plate (γ-hemolysis) and *S. aureus* ATCC 25923 had a clear zone of hydrolysis surrounding the colonies (β-hemolysis) (Figure 1F), indicating that it lacked hemolytic enzyme activity [29].

### 3.4. Antibiotics Resistance Analysis

Disk diffusion was used to evaluate antibiotic susceptibilities [11]. The sensibility to eight antibiotics (meropenem, amoxicillin–clavulanic acid, kanamycin, chloramphenicol, gentamicin, azithromycin, neomycin, and erythromycin) of the isolate was confirmed (Table 2) and the isolate was resistant to four antibiotics, and meanwhile, was intermediately sensitive to one. It seemed to be common to find resistant phenotypes in fermented foods. Several other studies found resistance to aztreonam, cycloserine, kanamycin, nalidixic acid, polymyxin B, and spectinomycin in 53 tested strains [30]. Velitchka found five *L. plantarum* strains resistant to ceftriaxone and ampicillin, a cephalosporin drug that damages the peptidoglycan layer [31]. However, LAB is deemed safe as long as they have no gene allowing horizontal transmission, and there have already been reports of antibiotic-resistant bacteria in various yogurt products on the market [32]. We then examined the presence of the resistance genes by PCR amplification to clarify whether the strain carried the resistance genes listed in Table 1. As the results elucidated, just the *gyrA* and *vanX* genes were found in the listed resistance genes while the 16S RNA bands as controls were clear. Mutations in the quinolone resistance determining areas of *gyrA* and *parC* lead to alterations in the topoisomerase (amino acid substitutions in the enzyme) together leading to quinolone resistance in *L. plantarum*, while *parC* was absent in our isolate thus having no effect [33]. For vancomycin, only the acquisition of at least three genes (*vanH*, *vanA* (or *vanB*), and *vanX*) combined into an operon can lead to this resistance mechanism, it is also an intrinsic resistance in fermentative lactic acid bacteria group [34]. In previous reports, the transfer of genes from *Lactiplantibacillus* to others seemed impossible [35], implying that the probiotics with *vanX* and *gyrA* also might be applied. These findings made us predicate the isolate virtually free of antibiotic transmissible concerns.

### 3.5. Acute Oral Toxicity Studies in Mice

**Figure 2:**
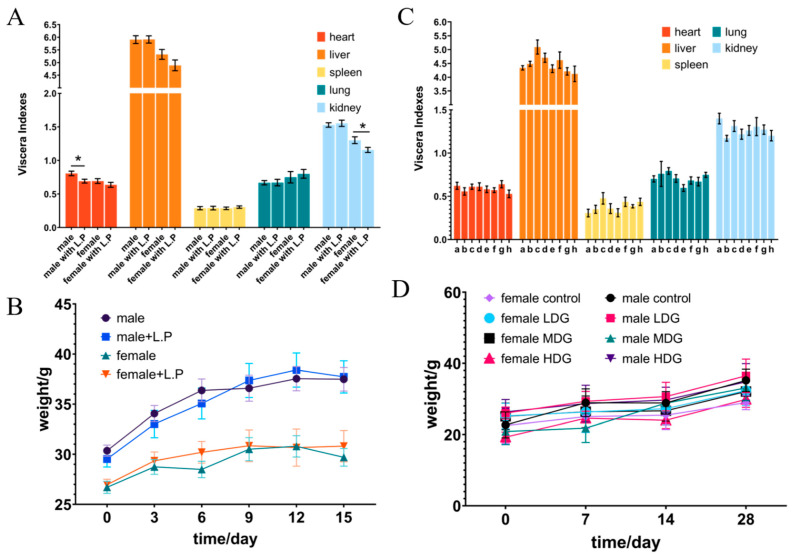
The changes in viscera index and body weight in mice administered with *L. plantarum* WYH by oral gavage for acute oral toxicity (**A**,**B**) and 28-day repeated toxicity (**C**,**D**). (**C**) a–d, respectively, represented normal saline (0), low concentration (10^8^), medium concentration (10^9^), and high concentration (10^10^) feeding groups of male mice. Additionally, e–h had the same means in the female mice group. * *p* < 0.05.

**Figure 3:**
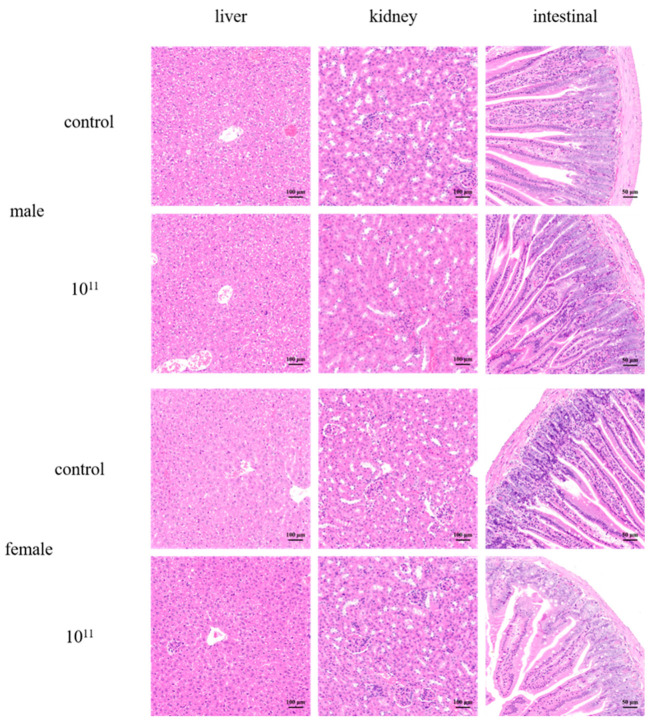
Light micrographs of the sections of the liver, kidney, and intestine of control mice, and mice have given a high concentration of 10^11^ CFU/mL of *L. plantarum* WYH. (×200).

After being fed with *L. plantarum* WYH, the mice had no abnormal changes in their skin, eyes, or mucous membranes; no abnormalities in body movement, behavior, or gait; no tremor, convulsion, drooling diarrhea, sleepiness, or other symptoms. The change in the body weight of mice is a direct reflection of their health. The body weight of mice was tracked at the same time on days 2, 5, 8, 11, and 14. (Figure 2B). During the entire treatment, there was no effect of oral administration on the body weight and mortality in both control and experimental groups. In preceding studies, the feeding of 10^10^ CFU/mL of LAB isolates did not influence body weight, general signs, and food consumption during the acute oral toxicity testing [28,36]. The serum biochemistry results in Table 3 showed that all groups exhibited normal ranges of hematological parameters [37]. Basophils, eosinophils, lymphocytes, monocytes, and neutrophils are white blood cells (WBCs) that mediate immune systems and defend the body against allergies, infections, and illnesses [38]. Analysis of hematological parameters can often suggest a visual overview of potential damage and provide a basis for clinical toxicity risk assessment [37], and our results showed that *L. plantarum* WYH did not trigger an inflammatory response. To explore histopathological damage to the organs, we then slaughtered and dissected the mice in the end. The heart, liver, spleen, lung, and kidney were harvested to calculate the viscera index. The results (Figure 2A) demonstrated there was no remarkable difference in liver, spleen, and lung weight between each group. A slight decrease in weight in the normal range in variation was observed in the heart in the male group and the kidney in the female group [37], which showed a significant difference (*p* > 0.05), while these differences were absent in appearance of the organ or other sex group, indicating no extreme damage was caused by *L. plantarum*. Acute toxicity studies may provide a preliminary reference to determine the appropriate dose and toxicity of the product. The intestine is the first organ to be exposed to the test substance, and the kidneys and liver are vital immune organs responsible for metabolism. With no difference in any of the organ indexes and the histological sections of liver, kidney, and intestinal organs were selected for HE staining to observe pathological changes. All these features could be concluded that abnormalities or histopathological changes in the organs, the liver, kidney, and intestine, were absent from necrosis, fibrosis, and loss of normal architecture in HE-stained tissue sections (Figure 3). The intestinal mucosa had a normal shape and structure, the intestinal villi were neatly arranged, the crypt was shallow and clear, and without detachment or separation between the intestinal epithelium and lamina propria in the normal group. The morphology of kidney tissue, capillary bulb volume, and the shape and structure of glomeruli were normal, and there was no obvious intracellular hyperplasia or inflammatory cell infiltration. The structure of hepatic lobules and nucleus pulposus tissues were intact without abnormal space enlargement, and the morphology of hepatocytes around the central vein of hepatic lobules was clear and well arranged. In the acute oral toxicity test of the substance, if there were no clinical changes of toxicity or mortality, then it can be initially considered non-toxic [37,39].

### 3.6. 28-Day Oral Toxicity Studies in Mice

**Figure 4:**
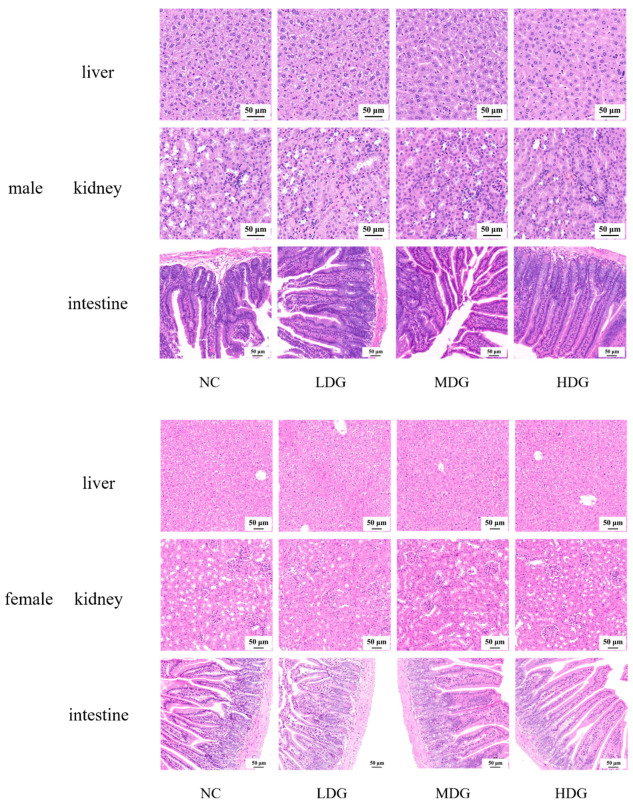
Tissue samples were collected for HE staining and pathological analysis was conducted. Liver, kidney, and intestine sections in mice with different concentrations of strain for 28-days repeated toxicity. The NC was fed with the saline and LDG, MDG, and HDG were fed with the saline containing 10^8^, 10^9^, and 10^10^ CFU/mL of isolate, respectively. (×200).

Based on the acute toxicity data, a 28-day repeated toxicity study was performed to further assess the safety of the isolate. In animal toxicity studies, feed consumption, body weight, and organ index are good indicators of the adverse effects of a substance, which visually reflect the influence of the tested animals [40]. There was no significant difference in the weight change (Figure 2D) and food consumption of the mice in each group. The potential damage to the major organs was analyzed after 28 days of therapy by calculating the ratio of each organ to body weight. The mice’s organ indexes showed no abnormal alterations (Figure 2C), implying no morphological changes in the organ. Hematological data and serum biochemical indicators are shown in Table 4. Hematological parameters are commonly used to determine inflammatory and infectious conditions, and apparently, the addition of *L. plantarum* WYH did not make a difference. To detect organ changes and reveal potential probiotic functions, serum biochemical parameters, and serum concentrations of alanine aminotransferase (ALT), aspartate aminotransferase (AST), glucose (Glu), urea, creatinine (CREA), and cholesterol (CHO) were measured. These features were used to evaluate glucose levels and the liver and renal functions [41,42], showing no marked changes, suggesting the isolate had no obvious effects on regulating blood glucose or energy metabolism. As in the acute toxicity experiments, we selected the liver, kidney, and intestine for further pathological analysis after confirming that there was no difference in the organ index of the vital organs. From Figure 4, we could find the hepatic lobule and cord structure were complete, the cells were firmly packed with distinct borders, and the cytoplasm was rich and evenly colored. Meanwhile, the nucleus was spherical and normal in size, and the inflammation was absent in liver tissue. In the renal tissue, several cortical glomeruli and tubular epithelial cytoplasm near the derm–myeloid junction were slightly loose and vacuolated, and no visible inflammation. In the mucosal layer of the small intestine, without separation between epithelium and lamina propria and lymphocyte inflammatory infiltration, the intestinal gland and connective tissue arrange regularly. No significant difference between the acute toxicity study and the 28-day repeated dose oral toxicity test, concluding that *L. plantarum* WYH is approximately safe in vivo. Studies of acute toxicity could provide preliminary toxicity data for determining appropriate doses and also assess the level of 28-day repeated-dose oral toxicity [36]. Additionally, 28-day repeated-dose oral toxicity study provides a preliminary evaluation of its effect on visceral function and more accurate data for further investigation because of its longer duration and repeatability [43].

## 4. Conclusions

The functional properties in vitro, antibiotic resistance, and toxicity in mice were investigated to assess the safety of *L. plantarum* isolated from Jiangshui. All the results successfully elucidated that the isolate was following basic safety principles of the international regulations and can be generally recognized as safe, providing valuable information for future functional development and application. Importantly, this study introduced a novel safe isolate for functional probiotic strains in the future.

## Figures and Tables

**Table 1:** List of primers used to detect the virulence genes.

Gene		Primer Sequences (5′ to 3′)
*16s rRNA*	27F	AGAGTTTGATCMTGGCTCAG
	1492R	CGGTTACCTTGTTACGACTT
*tetM*	F	GTGGACAAAGGTACAACGAG
	R	CGGTAAAGTTCGTCACACAC
*tetK*	F	TTAGGTGAAGGGTTAGGTCC
	R	GCAAACTCATTCCAGAAGCA
*tetL*	F	CATTTGGTCTTATTGGATCG
	R	ATTACACTTCCGATTTCGG
*tetS*	F	TGGAACGCCAGAGAGGTATT
	R	ACATAGACAAGCCGTTGACC
*bla*	F	CATARTTCCGATAATASMGCC
	R	CGTSTTTAACTAAGTATSGY
*vanA*	F	TTGCTCAGAGGAGCATGACG
	R	TCGGGAAGTGCAATACCTGC
*vanB*	F	TTATCTTCGGCGGTTGCTCG
	R	GCCAATGTAATCAGGCTGTC
*vanX*	F	TCGCGGTAGTCCCACCATTCGTT
	R	AAATCATCGTTGACCTGCGTTAT
*aac*	F	CCAAGAGCAATAAGGGCATA
	R	CACTATCATAACCACTACCG
*aph*	F	GCCGATGTGGATTGCGAAAA
	R	GCTTGATCCCCAGTAAGTCA
*strA*	F	CTTGGTGATAACGGCAATTC
	R	CCAATCGCAGATAGAAGGC
*strB*	F	ATCGTCAAGGGATTGAAACC
	R	GGATCGTAGAACATATTGGC
*aadA*	F	ATCCTTCGGCGCGATTTTG
	R	GCAGCGCAATGACATTCTTG
*ant*	F	ACTGGCTTAATCAATTTGGG
	R	GCCTTTCCGCCACCTCACCG
*cat*	F	CATARTTCCGATAATASMGCC
	R	TTAGGTTATTGGGATAAGTTA
*gyrA*	F	CAMCGKCGKATTCTTTACGGAATG
	R	TTRTTGATATCRCGBAGCATTTC
*parC*	F	GCYTCNGTATAACGCATMGCCG
	R	GCYTCNGTATAACGCATMGCCG
*ermE*	F	CATTTAACGACGAAACTGGC
	R	GGAACATCTGTGGTATGGCG
*ermC*	F	CAAACCCGTATTCCACGATT
	R	ATCTTTGAAATCGGCTCAGG

The presence of antibiotic genes was highlighted in colors.

**Table 2:** Strains antibiotic susceptibility test.

Antibiotic	Inhibition Zone (mm) ^a^		CLSI Classification ^b^	
	*Lactiplantibacillus plantarum*	*Staphylococcus aureus*	Susceptible Zone (mm)	S-I-R
TE (30 μg)	18.24 ± 0.26	21.95 ± 0.09	≥19	I
MEM (10 μg)	29.67 ± 0.31	20.74 ± 0.12	≥16	S
AML (10 μg)	33.61 ± 0.23	17.45 ± 0.05	≥18	S
AMP (10 μg)	NA	16.12 ± 0.12	≥19	R
VA (30 μg)	NA	NA	≥15	R
S (10 μg)	NA	19.21 ± 0.05	≥14	R
CIP (5 μg)	NA	25.25 ± 0.18	≥21	R
K (30 μg)	10.59 ± 0.10	7.82 ± 0.01	≥18	S
C (30 μg)	20.61 ± 0.09	23.72 ± 0.13	≥18	S
GEN (10 μg)	17.13 ± 0.14	14.35 ± 0.14	≥10	S
ERM (15 μg)	27.96 ± 0.06	NA	≥23	S
NEO (10 μg)	17.22 ± 0.16	14.80 ± 0.02	≥16	S
AZI (15 μg)	19.15 ± 0.07	9.00 ± 0.08	≥18	S

^a^ Values are expressed as mean ± SEM from three independent experiments. NA means nonexistent circle. ^b^ Standard interpretation of antimicrobial susceptibility tests of enterococci with disc diffusion method in accordance with CLSI standards: S, susceptible; I, intermediate; R, resistant. TE: tetracycline, MEM: meropenem, AML: amoxicillin–clavulanic acid, AMP: ampicillin, VA: vancomycin, S: streptomycin, CIP: ciprofloxacin, K: kanamycin, C: chloramphenicol, GEN: gentamicin, ERM: erythromycin, AZI: azithromycin, NEO: neomycin.

**Table 3:** Hematological parameters in mice for acute oral toxicity.

Hematological Parameters	0		10^11^	
	Male	Female	Male	Female
White blood cells (10^9^/L)	3.69 ± 0.16	3.94 ± 0.06	4.20 ± 0.28	4.72 ± 0.39
Neutrophils (10^9^/L)	0.06 ± 0.01	0.25 ± 0.02	0.08 ± 0.01	0.45 ± 0.12
Lymphocytes (10^9^/L)	3.27 ± 0.23	3.67 ± 0.07	3.90 ± 0.46	4.26 ± 0.30
Monocytes (10^9^/L)	ND	ND	ND	ND
Eosinophilic (10^9^/L)	ND	ND	ND	ND
Basophilic (10^9^/L)	0.01 ± 0.005	0.02 ± 0.000	0.02 ± 0.010	0.02 ± 0.010
Neutrophil percentage (%)	11.23 ± 4.58	6.25 ± 0.64	7.60 ± 5.78	9.18 ± 2.32
Lymphocytes percentage (%)	88.25 ± 4.65	93.20 ± 0.56	91.95 ± 5.79	90.38 ± 2.34
Monocytes percentage (%)	0.08 ± 0.04	0.03 ± 0.03	0.03 ± 0.03	0.10 ± 0.06
Eosinophilic percentage (%)	ND	0.030 ± 0.030	ND	ND
Basophilic percentage (%)	0.45 ± 0.05	0.50 ± 0.11	0.43 ± 0.13	0.35 ± 0.06
Red blood cells (10^9^/L)	7.99 ± 1.23	10.49 ± 0.21	9.27 ± 0.84	9.92 ± 0.38
Hemoglobin (g/L)	151.00 ± 9.43	160.75 ± 4.27	157.25 ± 4.55	159.50 ± 3.40
Hematocrit (%)	38.78 ± 5.49	49.08 ± 1.33	45.75 ± 4.02	47.73 ± 0.57
Mean corpuscular volume (fl)	48.93 ± 0.82	46.73 ± 0.42	49.40 ± 0.60	48.25 ± 1.43
Mean corpuscular hemoglobin (pg)	15.88 ± 0.34	15.33 ± 0.18	17.33 ± 1.41	16.13 ± 0.34
Mean corpuscular hemoglobin concentration (g/L)	324.50 ± 1.96	327.75 ± 2.53	350.25 ± 25.96	334.25 ± 3.35
Red blood cell distribution width (SD)	14.20 ± 0.090	15.30 ± 0.45	13.95 ± 0.33	13.45 ± 0.39
Red blood cell volume distribution width (CV)	26.98 ± 0.56	27.83 ± 1.02	26.80 ± 0.72	25.20 ± 0.91 *
Platelets (10^9^/L)	401.25 ± 65.39	320.25 ± 160.97	365.00 ± 203.01	403.00 ± 198.78
Mean platelet volume (fl)	7.13 ± 0.23	22.58 ± 15.81	7.20 ± 0.19	6.48 ± 0.19
Platelet distribution width (%)	15 ± 0.23	14.78 ± 0.11	14.85 ± 0.06	14.90 ± 0.11
Plateletcrit (%)	0.12 ± 0.04	0.26 ± 0.09	0.10 ± 0.02	0.25 ± 0.12

Each value was expressed as the mean ± SEM (*n* = 4). A significant correlation was noted by * *p* < 0.05 compared with controls for same-sex. ND means not detected.

**Table 4:** Hematological parameters and serum biochemistry in mice for 28-day repeated toxicity.

	NC	LDG	MDG	HDG
Hematological parameters				
White blood cells (10^9^/L)	6.98 ± 0.70	8.88 ± 0.61	5.26 ± 0.24	5.72 ± 0.61
Neutrophils (10^9^/L)	1.26 ± 0.28	1.18 ± 0.49	0.61 ± 0.22	0.68 ± 0.23
Lymphocytes (10^9^/L)	5.65 ± 0.62	8.36 ± 0.78	4.34 ± 0.59	7.50 ± 2.74
Monocytes (10^9^/L)	0.030 ± 0.010	0.030 ± 0.010	0.010 ± 0.00	0.010 ± 0.010
Eosinophilic (10^9^/L)	ND	ND	ND	ND
Basophilic (10^9^/L)	0.050 ± 0.010	0.070 ± 0.020	0.040 ± 0.010	0.040 ± 0.010
Neutrophil percentage (%)	17.95 ± 4.12	10.93 ± 3.37	13.55 ± 5.34	8.88 ± 2.62
Lymphocytes percentage (%)	80.95 ± 4.06	88.05 ± 3.14	85.55 ± 5.26	90.48 ± 2.60
Monocytes percentage (%)	0.40 ± 0.15	0.28 ± 0.11	0.15 ± 0.030	0.15 ± 0.050
Eosinophilic percentage (%)	0.030 ± 0.030	ND	ND	ND
Basophilic percentage (%)	0.68 ± 0.12	0.75 ± 0.27	0.75 ± 0.18	0.50 ± 0.070
Red blood cells (10^9^/L)	9.68 ± 0.14	9.89 ± 0.18	8.44 ± 0.99	9.66 ± 0.20
Hemoglobin (g/L)	144.50 ± 1.32	147.25 ± 3.28	129.75 ± 15.94	143.25 ± 1.55
Hematocrit (%)	45.33 ± 0.54	46.85 ± 1.09	41.63 ± 4.85	46.00 ± 0.72
Mean corpuscular volume (fl)	46.80 ± 0.64	47.38 ± 0.66	49.38 ± 0.050	47.60 ± 0.39
Mean corpuscular hemoglobi (pg)	14.93 ± 0.21	14.90 ± 0.070	15.35 ± 0.22	14.85 ± 0.18
Mean corpuscular hemoglobin concentration (g/L)	319.25 ± 1.80	314.25 ± 4.33	310.75 ± 4.31	311.50 ± 1.66
Red blood cell distribution width (SD)	17.08 ± 0.86	18.70 ± 1.50	16.88 ± 0.65	17.08 ± 0.57
Red blood cell volume distribution width (CV)	33.00 ± 1.32	36.70 ± 3.26	33.88 ± 1.32	33.60 ± 1.33
Platelets (10^9^/L)	885.75 ± 98.30	626.25 ± 126.72	614.75 ± 121.37	1058.25 ± 110.94
Mean platelet volume (fl)	5.63 ± 0.090	5.88 ± 0.24	6.20 ± 0.11	5.68 ± 0.14
Platelet distribution width (%)	15.20 ± 0.070	15.08 ± 0.13	15.28 ± 0.060	15.15 ± 0.030
Plateletcrit (%)	0.50 ± 0.050	0.36 ± 0.070	0.38 ± 0.080	0.60 ± 0.050
Serum biochemistry				
ALT (U/L)	61.68 ± 5.97	74.65 ± 2.77	64.14 ± 6.65	61.08 ± 3.48
AST (U/L)	139.28 ± 5.76	163.15 ± 14.68	151.10 ± 3.40	139.90 ± 10.12
Glu (mmol/L)	6.35 ± 0.49	6.05 ± 0.345	6.95 ± 0.50	7.05 ± 0.41
Urea (mmol/L)	18.68 ± 0.61	20.76 ± 1.04	18.26 ± 2.38	20.51 ± 1.34
CREA (μmol/L)	26.29 ± 1.09	21.64 ± 0.14	24.19 ± 2.39	26.08 ± 1.89
CHO (mmol/L)	2.26 ± 0.12	2.33 ± 0.17	2.65 ± 0.12	2.40 ± 0.085

Each value was expressed as the mean ± SEM (*n* = 4). *p* < 0.05. ND means not detected. A significant correlation was compared with the NC group (fed with saline).

## Data Availability

The datasets generated and analyzed during the current study are not publicly available, but may be available from the corresponding author upon reasonable request.
